# The Second Annual Meeting of the Dental Protective Association of the United States

**Published:** 1891-02

**Authors:** 


The Second Annual Meeting of the Dental Protective Association, of the United States, was held at the Grand Pacific Hotel, Chicago, Dec. 16th, 1890. The President, Dr. Crouse, called the meeting to order and spoke as follows:

Our second year closes full of encouragement. Last year at our first annual meeting we had represented, in person and by proxy, 648 members. This, our second annual meeting is represented by nearly 1500 members showing that the membership has more than doubled during the year.

We have driven the Tooth Crown Company from Milwaukee where they had commenced suits against five members. Our attorneys entered a motion asking the Court to set and limit the time when all the testimony should be presented by the Crown Company. After hearing the arguments of the counsel on both sides, the Court limited the time to twenty days and before it was up the Tooth Crown Company withdrew all the suits at their own cost. A similar move in Baltimore, where six of our members had been sued, and we had taken charge of their suits, caused the Crown Company to withdraw all these suits at their own cost. This demonstrates the correctness of what we have all along claimed that the International Tooth Crown Company did not dare to enter into a fair contest as to the validity of their patents.

They have now commenced suits in New York and answers will be filed in due time. We accept licensees on same terms as others and afford them the same protection with the exception of a few licensees located in a very limited portion of the country.

The Dental Protective Association has been giving absolute protection to the entire dental profession while, thus far, less than one tenth of the whole number has joined our association.

We want every member of the profession to unite with us. Ten dollars is but a trifle for each dentist to pay for the protection and benefits he receives. Think of 15000 dentists in one association! Who can estimate the great good that such an organization can accomplish? Is it a dream? No! I expect to live to see it. It only depends on the amount of exertion the present 1500 members put forth. Let every member during the year get three new members. It will be an easy way to treble our membership which is the great work of the coming year.

We have saved the Dental Profession a million of dollars the past year and better still saved many of its members the humiliation of signing a license that robs a man of his manhood.

I want a committee appointed from this association, to examine our books and methods of doing the work.

On motion Drs. Gilmer, Fernandez and Ames were selected.

The election of a Board of Directors resulted in J. N. Crouse, T. W. Brophy and E. D. Swain being elected their own successors. After further routine business the meeting adjourned.

J. N. Crouse, President.
E. D. Swain, Secretary.

REPORT OF COMMITTEE.

After a careful examination of the management and accounts of the Protective Association of the United States, for the year to date, we have come to the conclusion that it is being managed carefully, economically and with good judgement.

We know positively that the chairman is giving to this work, much thought and time, which is the same as money to him; that during the year, he has attended several dental meetings, in the interest of the association; that he has devoted much time and labor to organizing the profession and in attending to its litigation, and that he has done all this entirely at his own expense and without one dollar of cost to the association.

The entire office expense of the association for the year amounts to ($240.00) two hundred and forty dollars and that for clerical help.

The Protective Association is saving hundreds of thousands of dollars annually to the profession, and we believe it to be the duty of every practicing dentist to assist its grand work by paying the membership fee and becoming identified with the association.

It is certainly unjust that a few should bear the entire expense for the benefit of the whole profession, and it is demoralizing to those who quietly accept the fruit of this injustice.

We would further recommend that, as soon as the management think it advisable, after due notice to the profession, the books be closed, and the protection of the association be withheld from non-members.

Thos. L. Gilmer

E. M. S. Fernandez } Committee.

W. B. Ames

